# Temporary Teeth

**Published:** 1871-11

**Authors:** Chas. E. Koch


*To be continued.*

### SELECTED ARTICLES.

### ARTICLE V.

### *Temporary Teeth.*

BY CHAS. E. KOCH.

Read before the Chicago Dental Society.

There is, perhaps, no subject in relation to the teeth, upon which the public is less informed than that which we are to consider this evening. Even from among the most enlightened of our patients upon other matters, we occasionally hear the remark, "I did not know it was of any importance to preserve the *baby* teeth, wont others come in their places?"

Possibly we are in a large degree responsible for this almost criminal ignorance; and we certainly mistake our calling, and are remiss in our duty, if we do not improve every opportunity to enlighten and educate the public mind upon this subject. We should always impress upon mothers and those having the care of children, the necessity of taking care of the deciduous teeth; and we certainly will find a higher reward in the contemplation of the results of such few words of instruction and admonition, that we could possibly realize in any other way.

While general rules may be of some service as a guide in practice, these can seldom be closely adhered to in any given case, as special conditions or contingences may demand a marked deviation in treatment of apparently similar cases.

To be able to treat deciduous teeth scientifically requires by far more circumspection and reflection than would a similar condition in the permanent organs. We will, in nearly all cases, have to exercise our individual judgments based upon physiological and pathological laws, and the anatomical relations of the deciduous teeth to their successors, before we can make a conscientious diagnosis, or prognosis.

An all-wise Providence has given to children a set of teeth, which the study of physiology has convinced us, are given not merely as an ornament, but for a functional purpose. These are to last for a certain time, when they are to be replaced by such others as will more readily perform the increased requirements of an increased organism. This fact ought to convince us that our efforts should be in the direction of preservation.

To this end all the remedies generally employed as preventives of caries should be recommended. Chief of these are cleanliness—of the body as well as the oral cavity—pure air and properly nutritious food.

Should caries occur in the deciduous teeth at an early age, and be allowed to progress, it would probably cause serious inflammatory disease in the surrounding tissues, and premature loss of these teeth. Hence such carious cavities ought to be filled as early as possible with some suitable material, with a view of preserving them during their supposed time of usefulness. The molars and cuspids being thus affected, before say the ninth year, should especially be treated in this manner. As these are generally succeeded by others in from the tenth to twelfth year, it is hardly necessary to attempt preserving them through this means, should caries set in after the times first mentioned, unless we have some good cause for suspecting a retarded, or numerically deficient, second dentition. Should we know that either of the child's parents suffered from a retarded second dentition or a deficiency in number of the permanent teeth we would have good cause for attempting to save the deciduous teeth beyond their usual time of shedding.

Unless the incisors are attacked by caries at a very early age, say the third or fourth year, it is hardly necessary to interfere with them, as caries can be more readily retarded in these teeth by especial care. If it be deemed advisable to interfere with caries in the teeth by an operation, it may often be found that cutting away the diseased portion by means of proper instruments, and leaving a polished surface, will most effectually prevent further progress of the disease, and preserve the teeth during their natural lifetime. The fact that these teeth are usually succeeded by the teeth of replacement, in from the sixth to eighth year, must not be forgotten. The process of calcification in the permanent teeth of this class commencing quite early, there is less danger of injury being caused to their germs from lesions upon their decidious predecessors, than in the case of those teeth having a later period of eruption.

Should periostitis occur in the deciduous teeth, the treatment ought to be the same as in the case of the permanent teeth.

If the pulp has become exposed in teeth which it is essential to preserve for a while longer, a filling of oxychloride of zinc should be introduced in the carious cavity, having previously treated this as we should have done in the permanent teeth. If we do not succeed in keeping the zinc filling dry sufficiently long to insure its hardening, we can remove all but a cap of it over the exposure and fill over this with Hill's stopping, gutta percha, or if you please, amalgam, well washed and pressed. I must declare my preference for the first named substance.

It has been for a long time a matter of doubt in my own mind, whether the heroic practice of using arsenious acid in deciduous teeth, for the purpose of devitalizing their pulps, is judicious or not. Since the almost uniform success with oxychloride of zinc there is hardly any excuse for incurring the risk which almost in all such cases must accompany the use of arsenic. No matter how astute our observations may be we cannot tell to what extent absorption of the roots has progressed, and hence we cannot tell how far the action of the arsenic may penetrate, even with as much certainty as in the case of the permanent teeth; and you will no doubt bear me out in the assertion that even in these teeth this *certainty* is *doubtful.* We have often to extract deciduous molars with divergent roots, which remain quite firmly fixed and which would have led us to suppose that absorption had not pro gressed, far; yet, upon removing these teeth, we find that the dentinal substance next to the base of the pulp is entirely gone, and the pulp in contact with the plexus of absorbents. Can we tell how much of the tender tissues, besides the pulp would be destroyed by an arsenous application in such a case?

The permanent tooth may have advanced so far in its process of calcification as to escape injury, but its alveolus certainly would suffer. Necrosis and exfoliation of this latter migh ensue; and may not the sequestrum, in trying to make its exit, exercise sufficient force to misdirect the advancing permanent tooth, so as to cause malposition of this in the dental arch?

A lady patient, while absent from the city, suffered with severe pain in an upper left cuspid tooth, which had been previously treated for alveolar abscess through an opening to the dental canal, made from the anterior cervical surface of the tooth.

The dentist upon whom she called, evidently did not understand the case, and made an application to *destroy the nerve* through this opening, which of course did not afford her any relief. She returned about a week after, and I then found the labial side of the gum in a very much inflamed condition, and observed spiculæ of the alveolar process quite loose, portruding between the affected tooth and its anterior and posterior neighbors. These were removed, and the tooth treated with sedatives and stimulants until the pain had entirely ceased, and its surrounding tissues presented the appearance of health. The patient was not seen again for over three years, until very recently when I found that the labial plate of the alveolar socket was entirely gone, as also the soft tissues over it. The root is entirely denuded of its covering, and is a black unsightly looking mass. The tooth is still quite firmly fastened, but seems to have made an advance anteriorly, being now more prominent than its mate. This is probably caused by new deposits of bone upon the inside of the paltal plate of the socket. This case does not properly belong here, unless it may aid us to judge by analogy.

After the exhibition of arsenic in a deciduous tooth, injury to the alveolus may not be so permanent, as the growth of the alveolus of the permanent teeth is contemporaneous with their eruption, and is not entirely completed until some time after these teeth have assumed their permanent position; still, as we cannot tell just in what manner, and to what extent this drug may act injuriously, we had much better abstain from its use altogether.

Whether the premature loss of the deciduous teeth predisposes irregularity in the permanent teeth or not, seems still to be a mooted question. Mr. Tomes thinks there is more harm done by extracting too late than too early, as the now absorbed deciduous roots may so much resist the advance of the permanent teeth as to cause them to take position posterior or anterior to their deciduous predecessors. Prof. Harris thinks much harm is done by premature extraction, and a consequential bony contraction, and is diametrically opposed to Tomes. Before concluding which view to adopt, let us examine into observed physiological facts. Nearly all writers agree that the space occupied in the jaw anteriorly, by the ten teeth of the deciduous set, and the anterior ten teeth of the permanent set, is very nearly equal.

I have had imported from the publishers at Leipzig, a recent German work on the Pathology of the Teeth, by Prof. Weld, of Vienna, a notice of which I have seen in the *Cosmos* for February, of the current volume. The first seventy pages of this work are devoted to physiology and anatomy. Glancing over this, I noticed a series of measurements in regard to the growth of the jaw, which I deemed of interest enough to translate. With your permission, I will interline this translation here.

"Hunter was the first to take the position that the maxillary portions occupied by the deciduous teeth, do not grow after the completion of the first dentition. Fox agreed with him in all essentials. They arrived at this result by means of measurements of macerated jaws. Delebarre, upon the contrary, has tried to establish the longitudinal growth of the bone after first dentition, by clinlcal observations; he maintains that the deciduous teeth separate from one another at the age of five or six years, and says that individuals with whom this does not occur, are threatened with an irregular second dentition. Fox recognized this appearance before Delabarre, yet did not attach to it the same signification, as he believes that the anterior portion of the maxilla undergoes scarcely more than a change of form, which is in conformity to the shapes of the permanent teeth and does hardly add to its size. Thomas Bell remarks emphatically that we must not rely upon comparisons of jaws of different individuals. The only way to arrive at the truth in the matter is to examine the same jaw at different stages and compare the results. This says Bell, I have repeatedly done, and am not at all backward to say, that the ten anterior permanent teeth form a somewhat larger arch than the temporary teeth which preceded them. Chapin Harris joins in the belief of Bell, and thinks that the transverse and perpendicular dimensions of the anterior portion of the jaw, continue to grow until the completion of second dentition and even up to adolescence. Tomes inclines to the opinion of Hunter, and expresses himself as opposed to an interstitial bone growth.

According to Hunter, the growth of the arch of the lower jaw in its perpendicular dimensions, is owing to periosteal and in its transverse dimensions to expansive bone growth. He is of the opinion, that there does not occur any more essential growth in the arch of the lower jaw, between the first molars, after birth. Welker's measurements are said to agree with this. Generally Hunter expresses himself as opposed to an interstitial growth of bone. Rich. Volkman expressed himself in favor of this some time ago, and again recently. C. Ruge and Julius Wolff have also declared themselves in its favor. I join these for reasons which will become obvious in the perusal of this work.

There can be no doubt that, as already stated, the expansion of the arch of the jaw is produced by the formation of new bone substance upon its facial wall, and resorption upon the lingual wall. By this process the front teeth are shoved in an anterior, and the posterior teeth in an external (buccal), direction.

This locomotion of the teeth is, however, only to be thought of, if, in the interior of the bone, resorption also takes place, as otherwise the teeth, being covered by new layers upon the facial wall of the jaw, finally would be nearer the lingual wall, which is not the case.

Hence there must be an interstitial resorption. Fox advocates a change of form of the jaw during gsowth, yet this is, as mentioned by him, only true in the case of the upper jaw. He says: if we compare the jaw of a child with that of an adult, we will remark a very apparent difference; that of a child forms nearly half of a circle, that of an adult half of an elipsis. This metamorphosis of the jaw we will investigate closer further on.

To establish the proportions of the growth of the lower jaw we must first search for the least variable point from which to commence our measurements. As a matter of course we cannot attain to absolute exactness, and the truth can only be approximately arrived at. The unavoidable errors in observation are weightiest when the distances to be measured are smallest. The longitudinal dimensions offer fewer difficulties in their establishment than do those of height and thickness.

I have instituted a series of measurements upon forty-five skulls of children in regard to the growth of the lower jaw lengthwise, and also partially with a view to become acquainted with the individual differences; and have used for this purpose a thin, somewhat moistened, strip of paper closely applied;—and with this I have measured the peripheral limits. Measurements made upon a crooked object by means of a pair of dividers, give, as a matter of course, their sector, and this latter remains the same under variable crooks. As a fixed point, for measurements of the anterior portion of the lower jaw, we take the mental foramen, but here we must remember that the locality of this hole is not a constant one. Its anterior border is sometimes found (upon the lower jaws of adults,) between the two bicuspids, sometimes immediately under the first, and sometimes under the second bicuspid, yes, even sometimes in a plane with the posterior canal surface of the second bicuspid. These variations occurring in a small number of lower jaws gives us a source of error of from three to four Mm.* We will not take any note of this, but keep in view the distance from the line of union of the two halves of the lower jaw (which is easily marked with a lead pencil) to the anterior

*A millimetre is the 25th part of an inch or two-fifths of a line.

border of the mental foramen. In a five month embryo, we obtained a distance of ten Mm. New born child, 12-13; in 4, 5, 6, 7, 8 and 9 months old children, 15-18 Mm. In 1, 2, 3, 4, 5 and 6 year old children, the distance remains nearly the same, 18-19 Mm.; only in four cases they reached 20-23 Mm. In a child of the age of 1 year, five months and twenty seven days, with erupted central incisors, 20; in one, one year ten months and ten days old with erupted first molars, 21; in one, five years old with a complete set of temporary teeth, 21; and in a child six years and one month old, with strong denture, 23 Mm. In 7, 8, 9, 10, 11 and 12 year old children, it varies between 22 and 24, and in comparative measurements of lower jaws of adults, between 23-25 and 29 Mm. If we overlook extreme cases, we will find the most important growth of this portion of the lower jaw in the first months after birth, and a stationary condition after the eruption of the deciduous teeth, but during second dentition we find an increse which at least amounts to three Mm. To meet the accusation that no regard has been had to the thickness of the facial wall, several measurements were taken after removal of the facial wall, there appeared however, as was to have been expected, no essential difference.

Further measurements, in the same manner, by means of a strip of paper, were taken on the facial side of the lower jaw along its whole length, and that is from the marked place of union of its halves, to the most prominent part of the condyloid process, which can be done so long as the latter does not protrude too much over the level of the alveolar ridge. This periperal line amounts in a five months embryo, to 40 Mm in one of sevev months to 43; in thenew born child from 45-52 Mm; increases up to the fourth month to 58, up to the seventh month to 62, up to the first year to 67, up to the end of the second year after the eruption of the first molar to 77, up to the fourth, fifth, and sixth year with complete deciduous set of teeth to from 78-85, and in a child seven years old with erupted permanent molars to 100 Mm. Beyond this age such measurements can not well be made. After subtraction of the anterior border of the mental foramen, we obtain the rate of increase of the posterior mass from the last named border to the condyloid process; and we obtain in this way an increase from 10-23 Mm. in the anterior mass. Hence the quotients are as 2.56 : 2.3. The difference arises on the account of the more extensive growth of the posterioı division of the jaw.

*To be continued.*

